# Surgery

**Published:** 1894-09

**Authors:** 


### SURGERY.

EDITED BY DR. F. W. McRAE.

### FRACTURE OF THE LOWER END OF THE RADIUS.

As ordinarily met with, fracture of the lower end of the radius is the result of "cross-breaking strain," consequent upon over-extension of the hand upon the forearm. Whatever its degree, displacement is due to the secondary action of the anterior radio-carpal ligament upon the fragment already dragged off from the radius; and it is in the majority of cases largely maintained by the binding force of an untorn dorsal strip of periosteum, as originally shown by Pilcher. Much has been written, and more said, respecting impaction of the dorsal compact edge of the lower end of the upper fragment in the cancellous upper surface of the lower fragment; and proof of its existence has been found in inability to properly adjust the fragments by traction upon the hand in the direction of the long axis of the forearm. But, unless it may be under very unusual conditions, a force sufficient to pin the upper fragment in the lower would be sufficient to, and would split the lower fragment into two or more pieces and consequently prevent impaction. The resistance to replacement by direct extension is, in the great majority of cases, probably in all, not in any fixation of the upper in the lower fragment, but in the untorn periosteal band already mentioned. Only when such band is absent because of complete laceration at the time of injury, or it has been torn by the dragging force exerted through the hand, can proper apposition be secured by such manipulation. But if instead of pulling upon the hand it be over-extended, brought, in other words, in the position in which the fracture occurred, any existing periosteal band is at once relaxed; and moderate pressure upon the upper end of the lower fragment is sufficient to carry this fragment into its proper place at times with a very audible snap. Little or no pain is produced, no anesthetic is required; the securing of proper apposition of the fragments is the work of a few seconds. Personally, I have never had any difficulty in thus adjusting a case coming under my care within twenty-four hours after receipt of the injury. If there is, as there certainly is at times, a fixation of the ulnar styloid process under, or in the posterior annular ligament, such process will be set free either by simple over-extension, or by such combined with rotation. Once properly apposed, there is practically nothing other than outside violence to produce a secondary displacement.

Proper adjustment (and the earlier it is effected the better) will prevent or greatly limit effusions, exudations and adhesions. The unfortunate result, at times observed, which has been attributed generally to age, to rheumatic or gouty habit, to too short or too long employment of splints, to improper conduct on the part of the patient, or carelessness on the part of the doctor, is commonly, very possibly always, due to improper adjustment at the time of the original dressing; the displaced lower end of the upper fragment making such pressure upon the palmar surface near the wrist as to interfere with both arterial and venous circulation, especially the latter, and that not only directly but indirectly through the induced nervous disturbance. Under such circumstances the ordinary treatment by splint and bandage only makes a bad matter worse, the vessels and nerves being compressed between bone and board. This is one of the few fractures in which the chief difficulty lies in securing, not maintaining apposition. Once secured, only the simplest after-treatment is really required; a band around the wrist perhaps to lessen the after-spreading due to separation of and later somewhat misplaced attachment of the inter-articular cartilage. In view of possible after-ligation, as professional opinion is now, it is wiser to employ some form of splint, plaster of Paris or other, never extending below the wrist line, and thus supporting the hand which should be allowed to hang free.—P. S. Conner, M.D., Cincinnati, in the *International Journal of Surgery.*

### SHALL FRACTURE AT THE LOWER END OF THE HUMERUS BE TREATED IN THE FLEXED OR EXTENDED POSITION?

The author favors the extended position, the advantages of which are as follows: 1. It permits of the best flow of circulation, and is the position best suited to combat excessive inflammation. 2. It permits of inspection of the whole limb, and permits of comparison with its unbroken fellow. 3. It is a position in which the dressing can be applied with ease and simplicity. 4. In this position, if either condyle is broken, we have a natural splint, by means of which the natural articular surface can be preserved. 5. It is the position in which we dress fractures of the condyles of the femur, in which we get perfect joints with full range of motion.

In the treatment of fractures at the lower end of the humerus, no manufactured splints are ever required. If he has a skilled assistant, the author usually holds the limb extended, and asks him to apply the dressing. Otherwise he places the limbs in the care of attendants, and applies the dressing himself. A first dressing should always make provision for swelling. Hence, he either applies cotton or an elastic substance above and below the point of fracture, or laps about it pieces of flannel saturated in lead water and laudanum. The patient is then placed in bed and the limb made to rest comfortably upon a pillow. The renewal of the dressings for the first week must be left to circumstances. If dressed dry and the pain rapidly subsides, the first dressing is often not disturbed for a week.

As soon as the swelling subsides sufficiently, a soft bandage is applied directly to the limb. The author prefers thin flannel, buttercloth or the thinnest, coarse-woven, cheapest muslin. In applying the bandage, he makes no reverses except at the top and bottom when he wishes to change the direction of the bandage. He only uses the spiral and lets the bandage take its own course. By degrees, as the bandage runs up and down the limb, the whole is covered in. This mode of applying the bandage is immeasurably superior to the spiral-reverse, which latter creases the skin and slips. When the limb is properly dressed with the spiral, the bandage alone makes an excellent splint. On completing the bandage, a glutinous paste may be rubbed into the outer layer, or adhesive plaster may be made to cover in the bandage. Such a dressing will permit of easy motion without displacement.

Usually after the swelling subsides, the patient will be able to rise and walk about, but the arm must be left hanging, and not bent and put in a sling. If the hanging arm is painful, the patient should again assume the recumbent or the semi-recumbent position.

As to passive motion, if begun too early or with too much spirit, the result will be to protract the cure. Only gentle passive motion in the latter half of the treatment will be conducive to early functional return. The complete range of motion is often delayed for months after the cure is effected.—OSCAR H. ALLIS, M.D., Philadelphia, in the *International Journal of Surgery.*

